# The Influence of Civilization and Social Life upon Women, as Viewed from a Medical Standpoint*Read before the Medical Association of Georgia, April, 1892.

**Published:** 1892-06

**Authors:** D. C. Hurt

**Affiliations:** Columbus, Ga.


### THE INFLUENCE OF CIVILIZATION AND SOCIAL LIFE UPON WOMEN, AS VIEWED FROM A MEDICAL STANDPOINT.*

BY D. C. HURT, M. D., COLUMBUS, GA.

Amid all the changes which are taking place in the physical world, human life bears its portion. Whether it be called evolution or change consequent upon improvement or disintegration of human blood and human life, the changes are manifest.

In the science and art of medicine and surgery, great and wonderful strides mark our present era. Whether, therefore, some changes we now discover and attribute to diseases alone may or may not be the outcroppings of inherent or acquired malnutrition, remains yet to be explained. Looking back over the history of our science for the past ages, we find that despite the real changes there are many that are imaginary and dependent upon the fashions of men's mind and reasoning.

Somewhere and somehow there has grown in the human life of females that element of temperament and disposition which brings about more or less of disease destructive to human happiness. While in the present day so much attention is being paid to the diseases peculiar to women, it is highly proper that we, as medical men, should dwell with serious thoughts upon the causes and conditions that bring about these results. It is the office of the physician to assidiously apply himself first, to preventing and, secondly, to removing as far as possible all maladies that this human flesh is heir to, but when we open our eyes fully to the condition of 30 per cent. of our women of the present day, the facts stare us in the face that these evils are wonderfully multiplied.

There is a law of our physical being under which we are created, by which our bodies are to be governed for the preservation of health, the maintenance of life and the propagation of our race. What we mean by this law or its application is where not only one organ performs its legitimate function, but where all work together in one harmonious manner. The

*Read before the Medical Association of Georgia, April, 1892.

infringments upon these laws are surely followed by deleterious effects upon the violators. The most sensitive constitutions and the most delicate organs of that constitution are those which yield first to the penalties thus inflicted. We find, therefore, that the female constitution being differently formed, is increased in its nervous sensibilities and thereby more readily yields to such influences as ignore these laws. We find also that the habits of women as we advance in civilization and society life bring them directly to ignore these fundamental laws to such an extent as to create physical deformities and break down their nervous constitution. The fruits of these violations are everywhere manifest, from the slightest degree of nervous disturbance to that giant form insanity, from the most trifling disturbance of the generative system to the most incorrigible and death-dealing contamination of that nature which distinguishes her sex.

Gynæcology existed in the dark ages of medicine, and truly but little was known and less demanded on that line until within our present century. It will not do for us to assume the false argument in this case that, then ignorance was bliss and it was folly to be wise.

A practical demonstration exists to-day by comparison of our savage and civilized women of America. While the Indian squaw is unacquainted with female diseases and broken down constitutions on account thereof, our cultivated and civilized women are growing more and more intolerant of that life which pertains to their special duties as females. The Indian squaw lives in her hut and performs manual duties and undergoes hard physical labor without any interference with her female nature and to her the act of parturation is looked upon without dread, carries with it but little pain and scarcely interrupts her duties for one day. Our civilized and cultured women, if forsooth they arrive at healthy womanhood and maturity, are liable at each turn of life to become a prey to some one or more of the thousand maladies that eventually brings them under the care of a gynæcologist. Why this change? What means the multitudes of sanitariums and hospitals for women which are wonderfully on the increase as far as civilization extends? What means this large percentage of our females that must be unsexed in order to extend their days? Shall we accept this as an argument against civilization, culture of mind and body and the study of arts and sciences?

The style of dress which exposes the body to the most inclement weather, producing reversions of cold and heat; waist being tightly laced by steels, the body is kept in such a state of contortion that deformities result and produces deleterious effects upon parturient women, as well as disturbs the function of the whole organism.

The pollution of mind which is developed from reading sensational novels too often beget a corruption of thoughts and desires which militate against these laws of nature and lead to practices and indulgences which drift farther and farther away from an obedience to nature's laws. As one of the outcroppings of such influences I may cite the case of murder which took place at the hands of a young woman a few months ago in the city of Memphis and which has been followed already by like cases. This girl was so infatuated with her love for her own sex as to conceive in her depraved mind the idea of being discarded by her. Again, the French writer Belo in his book "My Wife" relates the destruction of the happiness of two families by that strange infatuation of the love of one man's wife for the wife of another.

Having seen that woman was naturally endowed with physical constitution fitting her for all duties in her sphere and seeing that now it is with great difficulty she passes through the period of true womanhood without becoming a prey to some disease, we must assign a cause and if the cause or causes are rightly determined, then we, as medical men and guardians of life and health, ought not to leave undone anything that will aid in bringing succeeding generations back to nature's standard of health and vigor. Whether these duties require us to give attention to the physical, moral, mental and social care of the patient or all of these combined, we should be honest enough with ourselves and have consideration enough for humanity, and have pride enough in our profession to perform these duties well.

How are we in the habit of dealing with this question which relates to the health and happiness of our females? If a lady calls at the office of a physician and he is satisfied after thorough inquiry into her history and her present condition, is he not too often content to make his prescription, accept his fee and tell his patient to call again in a few days, ignoring the fact that some good and wholesome advice pertaining to the management of her system and due care in her social habits, taking of proper food in proper quantities, retiring at healthful and systematic hours, would conduce more to restoring her health than would the prescription alone? In these days when parents are over-indulgent it is often the case that when a girl leaves the nursery with a depraved nervous system, vitiated appetite and a nature too highly sensitive to maintain health with her surroundings, she approaches puberty with her system already unfitted for functions yet to be established. She soon finds that her duties at school make her morose or irritable and that her mind is not capable of receiving readily the impressions sought, nor her body capable of enduring the fatigue made in search of them; she at once falls into the hands of a physician who finds already present depletion of the system, impoverished blood, nervous prostration, enfeebled digestion, constipation, amenorrhœa, etc. He begins with tonics, stopping the child from school and directing systematic exercise. He realizes the fact that to do his full duty in the case he must give the parents wholesome instructions depending upon the execution of which is the future welfare of the girl. In such a case as this the duties of prescribing and proscribing are each alike obligatory upon the physician who seeks to perform well his part as such; and the medical man who is well informed will have the satisfaction in after years of having dealt honestly with his patients.

Again, the girl who is about reaching puberty is rarely ever informed as to the care necessary when anticipating such an event in life, and often, by imprudence and ignorance combined, is at the very onset of womanhood, divested of health and invested with such diseases as entail lifelong suffering. In such cases the family physician will not be overstepping the bounds of propriety in advising the parents so that due caution may be exercised. For it is true that a large per cent. of the cases resulting from such causes and which we denominate suppressio-mensium, emansio-mensium, are produced at this period of life. The patient falls into our hands emaciated and hysterical, pleading for relief. Up to this time the physician has done well by prescribing medicine and hygiene, but he now enters the field invaded by new diseases, induration, ovaritis, displacements, and menstrual disorders. It is at this point that the gynæcologist with his armamentarium appears upon the field and begins his work of investigation.

It is not my purpose in this article to discuss the diagnosis and treatment of diseases, but encourage such general oversight and management as shall prevent rather than cure.

Drawing again upon our comparison between the savage and civilized nations, we cannot deny the fact that the discovered cause or causes lay within the range of prevention. These causes as we have seen are exerted upon our females at every age of life, upon the child as she leaves the nursery, upon her again as she enters school, and again upon performing school duties in approaching the age of puberty; while she is a young lady devoting herself to society and society life. Upon proper care at these ages our girls may be brought up with a union of the physical, moral and mental forces so combined as to avoid many diseases and render her capable of happiness and entail the same upon the healthy offspring.

By way of disgression, here allow me to allude to our schools of the present day. After years of careful observation, I am fully persuaded that our system of education militates greatly against health and healthy development of our girls. I have observed as have many of you, the strain upon the nervous system caused by an improper lighting of the study room; as through the medium of the optic nerve so affected, the whole system is often disturbed; also the baneful influence of bad heating and ventilation of school rooms impairing digestion and the proper growth and development of the body. While my connection with these schools as trustee has led me to these observations, I am glad to say that more interest is taken now than ever before both by teachers and trustees looking to the remedying of these evils. A vast amount of literature written by men of experience is being disseminated throughout the entire school system of this country, which promises to be productive of much good to future generations. Again, for the past three decades, it is a fact in statistics that our Southern families are not as productive as before and there is a growing disposition to antagonize nature's laws so far as they relate to procreation. The small number of children found in New England fifty years ago showed a marked contrast with the birth rate of the English, Irish and German. I fear that the mania for exemption from this legitimate duty has infested our Southern women and they too are drifting into disease and decay on account thereof. The proud queen of England deserves much more honor to her Royal Highness in the fact that she brought forth, nursed and trained her own children and thus has placed before her subjects an example worthy of imitation. Could women be convinced that their lives were in jeopardy upon any deviation or violation of nature's laws, we would have much less need for our sanitariums and the practice of gynæcology.

Resuming, I repeat that prevention of such interference is at once the duty and triumph of our art; the striving after this end, the highest function of medicine. The subject for our protection, the culmination of man's happiness—the most lovable and attractive in true and healthy womanhood.

The time may have been when physcians, lacking in the present information that we have this day attained, not over crowded with work and exceedingly jealous in the profession, would have decried any efforts tending to these higher duties of life in the prevention of diseases; but in this age of our science we find those who are best informed the strongest advocates for the proper rearing and development of our physical nature, who concede the necessity of a due regard to the moral and mental culture as well. That physician succeeds best and grows most whose broad philanthropic mind divests him of narrow and contracted views and allows him to reach out for the greatest possibilities of good to humanity. Our object and aim in prosecuting our science should be to carefully guard every element of the human nature, that there may be a uniform and co-ordinate development of all our faculties. With this purpose steadily in view and the practice constantly maintained, we would not be surprised that on the advent of the next century, forces and influences should be put in operation that would insure for succeeding generations heathfulness of mind and body, longevity and a re-establishment of that endowment to our nature which was originally given by an all-wise Creator.

Then let us begin that revolution which looks with all its combining influences and co-operative mediums to pulling down our hospitals, stamping out diseases and restoring our females to their wonted health, strength, and beauty. Let us, by wise counsel prudently given to parents, help them to govern their children, control their appetites to the use of proper diet, root out pernicious habits of dress, train their minds to digest healthful and enlightening literature, cultivate social and moral habits and thus ward off the miasmatic vices which are ever lurking just outside the nursery and seeking every opportunity to obtain its victim.

Let us call upon the co-operative efforts of those who have the custody of our schools and ask them to demand a strict observance to the rules of hygiene; to bear in mind the fact that the success of instructors depends very largely upon the maintenance of sound bodies in their pupils. Let us, as medical men, lose no opportunity as we come in daily contact with the people, to instill into their minds ideas on every line conducive to health. It is not improbable nor unreasonable for us to expect that ere the dawn of the 20th century our science shall be able to effectually stamp out many of the epidemics which now run rife over our towns and cities, often depopulating large families. It is not unreasonable for us to look for a Jenner or a Pasteur who shall save us from the ravages of yellow fever, diphtheria and a host of other ills that occasionally invade our land and country. With our present knowledge of antiseptics, we destroy those bacteria which lie broadcast about our homes, and arrest the germ-producing process already at work in the consumption of our bodies, and we should with equal dexterity and confidence in our skill, remove the cess pools of moral corruption and elevate the mind to higher functions and duties of life, making void the caverns of sin which pander to depraved appetites, thereby guarding us against those depravities of our nature which tend to foster in us vitiated blood, whose domain it is to contaminate our whole being. We should guard our commonwealth against the concealed viper discovered only by medical men, which lies ever and anon in our pathway seeking anxiously every opportunity of injecting its venom into the moral and social veins of human life, thereby leaving in us or in our systems that poison which is to be handed to our posterity as a defiled inheritance.

We should warn the people to guard against a Nemesis which stands with uplifted dagger ready to plunge into our bodies and suddenly snatch us out of existence. Let us appeal to our various governments—municipal, State and national—to co-operate with us both by their moral and financial influence, that they may aid us in removing the lepers beyond the reach of contamination, and through their sanitary laws aid us in building bulwarks over which no invaders dare attempt an ascent.

Finally, let us hope that every man, woman and child shall be so invested with the love of purity of life and all that is elevating, ennobling and refining, that sin and its votaries, which means but the pasture in which diseases are well-bred, shall find but a barren waste and perish thereon.

